# Effect of delayed sample draw after blood collection for haemoglobin test in South Korea

**DOI:** 10.4102/ajlm.v12i1.2008

**Published:** 2023-03-28

**Authors:** Hyerim Kim, Jongmin Kim

**Affiliations:** 1 Department of Laboratory Medicine, Pusan National University Hospital, Busan, Republic of Korea

**Keywords:** haemoglobin, blood specimen collection, erythrocyte count, specimen handling, phlebotomy

## Abstract

Between April and May 2022, 10 healthy adult non-patients were recruited from Pusan National University Hospital. Venous blood drawn into a syringe was transferred into test tubes with a zero-to-45-minute delay. The transfer was done sequentially in two positions with the syringe and the needle adaptor end (1) heading downwards and (2) heading upwards. Haemoglobin levels gradually increased over time in position 1 transfer while they gradually decreased in position 2. Therefore, blood must be transferred quickly from a syringe to a tube for reliable test results.

**What this study adds:**

Our findings confirm that delays between blood collection and transfer can affect haemoglobin levels.

## Introduction

Blood collection is necessary since numerous tests are performed using blood specimens. Inadequate blood sampling may lead to inaccurate results and mislead the clinician; therefore, collecting blood according to best practice guidelines is essential. Current guidelines presented by the World Health Organization, Clinical and Laboratory Standards Institute, and European Federation of Clinical Chemistry and Laboratory Medicine-Latin America Confederation of Clinical Biochemistry provides evidence-based recommendations for the stepwise process of adequate blood sampling.^1,2,3^ However, there is no detailed guidance on the time interval between collecting blood in the syringe and transferring blood to the test tube when using the needle and syringe system. Since these guidelines recommend using vacuum extraction instead of the needle and syringe system, there seems to be no need for guidance on transferring blood to the test tube. Nevertheless, the needle and syringe system is most commonly used in blood sampling,^2^ and delay in transferring blood to the test tube following sampling can occur, which may alter the results of blood tests, for instance haemoglobin levels. However, to the best of our knowledge, studies on the effect of delayed blood transfer to the test tube after phlebotomy on haemoglobin level results are lacking.

We were asked to evaluate the effect of delayed blood transfer from the syringe to the test tube after receiving complaints of erroneous haemoglobin level changes in several patients (Table 1). The physicians observed unanticipated changes in haemoglobin, such as a significant decline in haemoglobin in the absence of relevant events such as bleeding, surgery, or trauma. An incorrect result of haemoglobin level may mislead the clinician in monitoring the patient’s overall health and diagnosing haematologic diseases, including anaemia. The errors can be divided into pre-preanalytical, preanalytical, analytical, postanalytical, and post-postanalytical phases.^4^ Up to 68% of errors are pre-preanalytical,^4^ including the well-known sample quality factors: haemolysis, cryoglobulin, lipaemia, and clotting issues.^5,6^ Therefore, possible errors in all test phases were carefully examined. After excluding all potential causes of error, we found that all reported cases were sampled using the needle and syringe system and assumed that a delay between blood collection and blood transfer to the test tube may have resulted in a change in haemoglobin levels. This assumption is based on the hypothesis that the sedimentation of red blood cells occurred during the prolonged storage of sampled blood in syringes. Red blood cell sedimentation is well known, as in the erythrocyte sedimentation rate (ESR) test.^7^ However, red blood cell sedimentation in a phlebotomy setting and its effect on haemoglobin test results is understudied. Thus, we conducted this study to evaluate the effect of delayed blood sample transfer on the haemoglobin level by analysing samples with different time delays under two conditions: drawing blood from the sedimented bottom portion or the non-sedimented upper portion of the syringe.

**TABLE 1:** Reported cases of erroneous haemoglobin levels, South Korea, April 2022 - May 2022.

No. case	Gender/Age	Baseline Hb		Reported Hb		Re-test Hb† (Level [g/dL])
		Date	Level (g/dL)	Date	Level (g/dL)	
1	M/45	23 September 2021, 13:54	7.2	24 September 2021, 6:44	3.6	6.8
2	M/58	05 September 2021, 6:18	7.8	05 September 2021, 17:17	4.7	8.6
3	F/61	29 August 2021, 6:09	8.0	29 August 2021, 23:20	5.1	7.0
4	F/68	18 June 2021, 8:05	8.2	21 June 2021, 6:03	3.6	7.8
5	F/67	10 September 2021, 8:54	9.3	13 September 2021, 5:41	3.0	10.0
6	F/63	17 July 2021, 7:13	9.5	18 July 2021, 8:22	3.0	8.8
7	M/38	13 August 2021, 7:53	10.5	14 August 2021, 8:09	3.1	10.7
8	M/81	14 April 2021, 14:04	10.6	07 July 2021, 18:34	19.8	10.1
9	F/77	19 August 2021, 8:03	10.7	29 August 2021, 21:37	6.9	9.9

Hb, haemoglobin; F, female; M, male.

† , All retests were performed within 3 h of the initial (reported) test.

## Methods

### Ethical considerations

An application for full ethical approval was made to the Institutional Review Board of Pusan National University Hospital. The ethics approval number is 2204-004-113. All procedures performed in studies involving human participants were in accordance with the ethical standards of the institutional or national research committee and with the 1964 Helsinki Declaration and its later amendments or comparable ethical standards. Written informed consent was obtained from all individual participants involved in the study. The collected personal data was saved on a password-protected computer that only the authors had access to.

### Participant recruitment and data collection

Between April 2022 and May 2022, 10 healthy adult non-patients were recruited at Pusan National University Hospital, Busan, South Korea, using a recruiting poster. The volunteers were eligible for inclusion if they were aged between 20 and 60 years and weighed over 50 kg. Each participant was asked a series of prepared questions to determine their eligibility and collect information about them, such as their gender, age, and medical history. Volunteers were excluded if they were pregnant, had a history of a blood clotting disorder, or had inadequate venous blood collected.

### Blood collection

**FIGURE 1:**
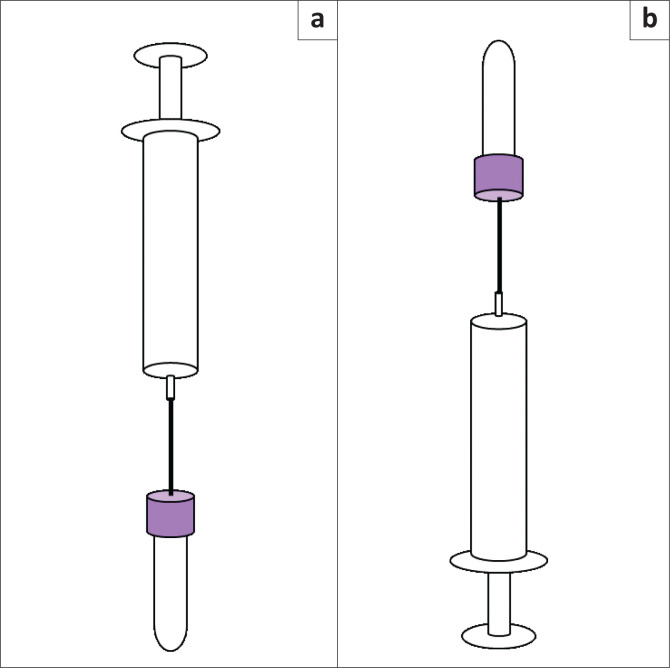
Illustration of needle and syringe positioning during blood transfer, South Korea, April 2022 – May 2022. (a) Position 1, syringe vertically positioned with needle adaptor end heading downwards. (b) Position 2, syringe vertically positioned with needle adaptor end heading upwards.

A trained laboratory technician collected blood from the veins in the antecubital fossa, once in each arm. First, blood was collected using a vacuum extraction system (multiple sample blood collection 21G needle and quick release needle holder, Becton, Dickinson and Company, New Jersey, United States). Next, 3 mL of blood for the K_2_ ethylenediaminetetraacetic acid (EDTA) tube and 2.7 mL for the sodium citrate tube (Becton, Dickinson and Company, New Jersey, United States) were collected, which we defined as control samples. On the opposite arm, 3 mL of blood was collected using a needle and syringe system with a 24G winged butterfly needle (JMS(K) Medical Supply, Seoul, Republic of Korea) into ten 10 mL syringes (Jung Rim Medical Industrial, Chungcheongbuk-do, Republic of Korea). The winged butterfly needle was used instead of the original needle connected to the syringe to minimise the number of venepunctures performed per participant. The syringes were placed horizontally on the ground without assistive devices. One mL of blood was transferred into two 3 mL K_2_ EDTA tubes at 0, 5, 10, 15, 20, 25, 30, 35, 40, and 45 min in two positions in the following order: (1) syringe vertically positioned with needle adaptor end heading downwards and (2) syringe vertically positioned with needle adaptor end heading upwards (Figure 1). These EDTA tubes were defined as the test samples.

### Outcome assessment

The activated partial thromboplastin and prothrombin times were determined using sodium citrate samples in CS-5100 (Sysmex Corporation, Kobe, Japan), ESR in EDTA tubes using TEST1 (Alifax, Padova, Italy), and complete blood count in EDTA tubes using XN-9000 (Sysmex Corporation, Kobe, Japan). For test samples, complete blood count was examined, and the changes in haemoglobin levels were analysed to evaluate the effect of delayed blood transfer.

### Statistical analysis

The Kruskal-Wallis test was used to analyse haemoglobin levels over time. The Kolmogorov-Smirnov test and the Shapiro-Wilk test were adopted to test normality. All statistical analyses were performed by SPSS^®^ 22 for Windows (SPSS Inc., Chicago, Illinois, United States). A *p*-value of less than 0.05 was considered statistically significant. The line graphs were generated using Microsoft Excel, and illustrations using Microsoft PowerPoint (Microsoft Corporation, Redmond, Washington, United States).

## Results

The baseline characteristics of 10 participants, three male and seven female (Online Supplementary Table 1), show that none had comorbidities. The haemoglobin levels of the control samples were consistent with the 0 min sample of the test sample. In addition, the control samples’ prothrombin times, activated partial thromboplastin, and ESR results were within the normal range except for those of two participants, which presented mildly elevated ESR. Among the control sample results, including ESR, activated partial thromboplastin, prothrombin times, and international normalised ratio, none seemed to be correlated with the amount of haemoglobin change over time.

**FIGURE 2:**
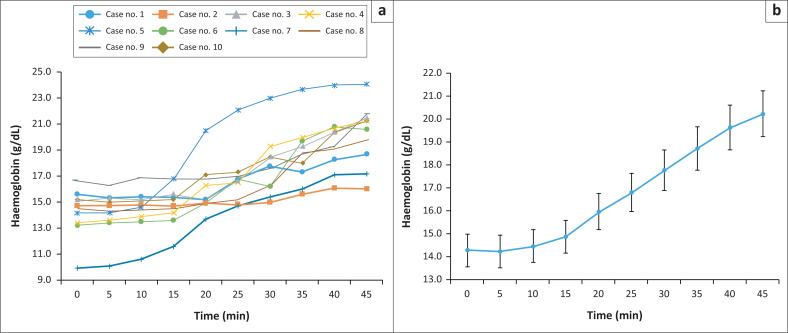
Changes of haemoglobin level (g/dL) over delayed blood transfer in position, South Korea, April 2022 – May 2022. (a) Represents the 10 cases of position 1, and (b) shows the increase of average haemoglobin levels of 10 cases (*p* < 0.001, Kruskal-Wallis test). Statistically significant changes between 0 min and 35 min (*p* = 0.006), 0 min and 40 min (*p* = 0.001), 0 min and 45 min (*p* < 0.001), 5 min and 35 min (*p* = 0.005), 5 min and 40 min (*p* = 0.001), 5 min and 45 min (*p* < 0.001), 10 min and 35 min (*p* = 0.011), 10 min and 40 min (*p* = 0.01), 10 min and 45 min (*p* < 0.001), 15 min and 35 min (*p* = 0.05), 15 min and 40 min (*p* = 0.008), and 15 min and 45 min (*p* = 0.002) were observed in the post hoc test.

The results of samples transferred while the needle adaptor end faced downwards had an increase in haemoglobin levels with time (Figure 2, *p* < 0.001). The average haemoglobin level increased from 13.9 g/dL at 0 min to 20.2 g/dL at 45 min. Compared to the 0 min sample, the changes appeared to be statistically significant at 35 min (*p* = 0.006), 40 min (*p* = 0.001), and 45 min (*p* < 0.001).

**FIGURE 3:**
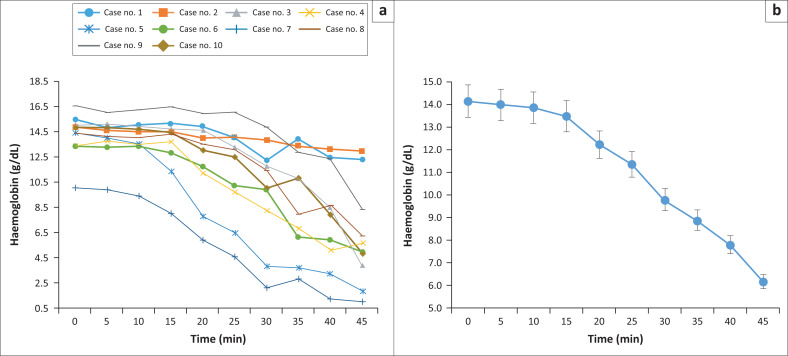
Change in haemoglobin level (g/dL) over delayed blood transfer in position 2, South Korea, April 2022 – May 2022. (a) Represents the 10 cases of position 2, and (b) shows the decrease of average haemoglobin levels of 10 cases (*p* < 0.001, Kruskal-Wallis test). Statistically significant changes between 0 min and 35 min (*p* = 0.033), 0 min and 40 min (*p* = 0.006), 0 min and 45 min (*p* = 0.001), 5 min and 40 min (*p* = 0.013), 5 min and 45 min (*p* = 0.001), 10 min and 40 min (*p* = 0.02), 10 min and 45 min (*p* = 0.002), and 15 min and 45 min (*p* = 0.008) were observed in the post hoc test.

Gradual decreases in haemoglobin levels were observed in samples transferred with the needle adaptor end facing up (Figure 3, *p* < 0.001). The average haemoglobin level dropped from 14.2 g/dL at 0 min to 6.2 g/dL at 45 min. When compared with 0 min, the changes appeared to be statistically significant in 35 min (*p* = 0.033), 40 min (*p* = 0.006), and 45 min (*p* = 0.001).

## Discussion

The findings of this study confirm that the interval between blood collection and blood transfer can affect haemoglobin levels. Regardless of needle position, the delay between the blood draw and the blood transfer led to incorrect haemoglobin levels. When the syringe was positioned vertically with the needle adaptor end heading upwards when transferring, the haemoglobin levels dropped with time. On the other hand, the haemoglobin levels increased with time when transferring blood with the needle adaptor end heading downwards. We believe that the transfer delay may have induced sedimentation of the sample transferring the supernatant, that is, needle adaptor end heading upwards, resulting in low haemoglobin levels and transferring the lower part of sedimentation, that is, needle adaptor end heading downwards, results in increased haemoglobin levels.

### Limitations

There are several limitations. Firstly, as all participants were between 31 and 41 years, the study samples may not represent the general population. The previously reported erroneous cases (Table 1) were relatively older patients, aged from 38 to 81. Secondly, blood transfer with the needle pointing downwards was performed after transferring blood with the needle pointing downwards; thus, the results of the former position may have been affected by the inversion. Thirdly, we used a 24G butterfly needle instead of the original 21G needle connected to the 10 mL syringe, which is one of our institution’s general settings. Therefore, the difference in needle gauges can be associated with erroneous factors, such as haemolysis.^8,9^ However, all 10 samples were obtained in the same position with the same needle. The needle change divulges the effects of delayed blood transfer. Lastly, in this study, only 1 mL of blood was transferred into 3 mL EDTA tubes to minimise the amount of blood sampling per participant. However, a study reported that acceptable complete blood count values could be obtained with as little as 1 mL of blood in 4 mL K_2_ EDTA tubes.^10^ Therefore, we believe that the method of this study using 1 mL of blood in 3.0 mL K_2_ EDTA tubes can be considered relevant.

### Conclusion

In conclusion, the findings of this study show that delayed blood transfer after venepuncture may lead to changes in haemoglobin levels; thus, blood should preferably be transferred directly into test tubes after collection.
